# Validity of Acute Cardiovascular Outcome Diagnoses Recorded in European Electronic Health Records: A Systematic Review

**DOI:** 10.2147/CLEP.S265619

**Published:** 2020-10-14

**Authors:** Jennifer Davidson, Amitava Banerjee, Rutendo Muzambi, Liam Smeeth, Charlotte Warren-Gash

**Affiliations:** 1 Faculty of Epidemiology & Population Health, London School of Hygiene and Tropical Medicine, London, UK; 2 Institute of Health Informatics, University College London, London, UK

**Keywords:** validation, myocardial infarction, heart failure, stroke; routinely collected health data

## Abstract

**Background:**

Electronic health records are widely used in cardiovascular disease research. We appraised the validity of stroke, acute coronary syndrome and heart failure diagnoses in studies conducted using European electronic health records.

**Methods:**

Using a prespecified strategy, we systematically searched seven databases from dates of inception to April 2019. Two reviewers independently completed study selection, followed by partial parallel data extraction and risk of bias assessment. Sensitivity, specificity, positive predictive value (PPV), and negative predictive value estimates were narratively synthesized and heterogeneity between sensitivity and PPV estimates were assessed using I^2^.

**Results:**

We identified 81 studies, of which 20 validated heart failure diagnoses, 31 validated acute coronary syndrome diagnoses with 29 specifically recording estimates for myocardial infarction, and 41 validated stroke diagnoses. Few studies reported specificity or negative predictive value estimates. Sensitivity was ≤66% in all but one heart failure study, ≥80% for 91% of myocardial infarction studies, and ≥70% for 73% of stroke studies. PPV was ≥80% in 74% of heart failure, 88% of myocardial infarction, and 70% of stroke studies. PPV by stroke subtype was variable, at ≥80% for 80% of ischaemic stroke but only 44% of haemorrhagic stroke. There was considerable heterogeneity (I^2^ >75%) between sensitivity and PPV estimates for all diagnoses.

**Conclusion:**

Overall, European electronic health record stroke, acute coronary syndrome and heart failure diagnoses are accurate for use in research, although validity estimates for heart failure and individual stroke subtypes were lower. Where possible, researchers should validate data before use or carefully interpret the results of previous validation studies for their own study purposes.

## Introduction

Ischaemic heart disease and cerebrovascular disease have been the leading causes of death globally for more than 15 years.^1^ In Europe, cardiovascular disease (CVD) deaths and prevalence have decreased but remain substantial; in 2015 an estimated 85 million people had CVD including 11.3 million with new diagnoses.^2^

CVD determinants and outcomes research increasingly utilize electronic health records (EHRs). EHRs contain comprehensive longitudinal health data, extracted from primary and secondary care clinical systems, for large patient populations which provide cost-effective data for research. EHR data is mostly “structured” with diagnoses coded using, for example, the International Classification of Diseases (ICD) but can also be “unstructured” with anonymized free-text notes.^3^ EHR-based research predominantly uses structured data. As the primary purpose of EHR data collection is clinical, it is essential to consider the validity of the data’s use in research.

EHR use is widespread in Europe, where many countries have national healthcare systems, and several systematic reviews have previously explored the quality of specific European EHRs.^4^–^7^ Other systematic reviews^8^–^12^ have investigated the validity of CVD diagnoses in computerized health-related records, which included EHRs but mainly drew results from disparate claims-based systems. The previous reviews did not separate results for EHR and claims data, the quality of which may differ due to the differences in setup and collection rationale.

In our systematic review, we provide an up-to-date assessment of the validity of acute CVD diagnoses recorded in European EHRs. We defined acute CVD as heart failure (HF), acute coronary syndrome (ACS), and stroke. These high-burden conditions are key diagnoses commonly included in the composite endpoint of major adverse cardiovascular events (MACE) which is increasingly employed in both clinical trials and observational research studies.^13^ We investigated whether the validity of these diagnoses differed by subtype, definition, data source, reference standard, and study population.

## Methods

### Protocol and Registration

Our protocol was published in October 2019^14^ following the Preferred Reporting Items for Systematic Reviews and Meta-Analyses Protocol guidelines (PROSPERO registration number CRD42019123898).

### Eligibility Criteria

**Figure 1:**
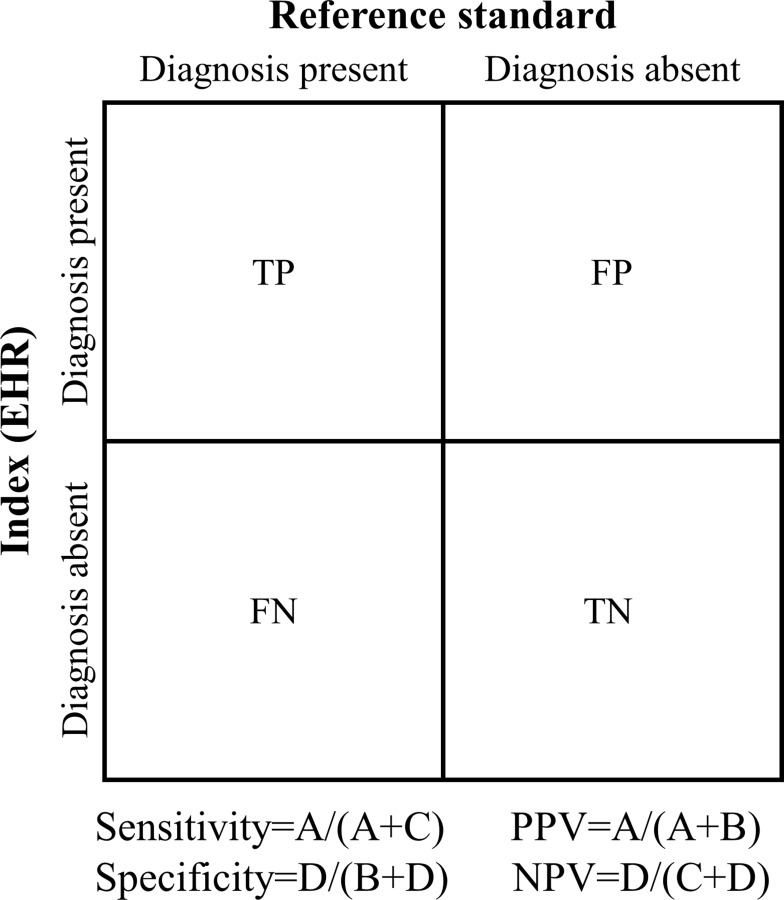
Illustration of validity estimates calculations.

We included articles that validated diagnoses in patients aged ≥16 years captured in any European primary or secondary care EHR. We excluded claims-based databases, disease registries, vital registration systems, or locally held databases. Articles needed to validate clinical codes for the diagnoses of HF, ACS, or stroke (Table 1) against a suitable internal or external reference standard. HF is most frequently a chronic condition which can deteriorate with acute exacerbations. HF may also have an acute onset, for example after an MI. The European Society of Cardiology (ESC) defines acute HF as rapid onset or worsening of symptoms and/or signs of existing HF.^15^ ACS encompasses different clinical forms of myocardial ischaemia which includes myocardial infarction (MI) and unstable angina. The specific diagnosis of MI or unstable angina depends on symptoms, signs, biomarkers, and ECG and/or autopsy findings, with the definitions refined over time.^16^ The diagnosis of stroke includes subtypes ischaemic stroke, intracerebral haemorrhage (ICH), and subarachnoid haemorrhage (SAH).^17^ At least one validation estimate (Figure 1) or the raw data to calculate it was required.

**Table 1:** Example Clinical Codes Included for Stroke, Acute Coronary Syndrome and Heart Failure Diagnosis Definitions

Diagnosis	Subtype	ICD-10	ICD-9	ICPC
Acute coronary syndrome	Myocardial infarction	I21	410	K75
	Unstable angina	I20.0		
	Cardiac arrest	I46		
	Other acute heart disease	I24	411	
Heart failure		I50	428	K77
Stroke	Subarachnoid haemorrhage	I60	430	K90
	Intracerebral haemorrhage	I61	431, 432	
	Cerebral infarction	I63	433, 434	
	Non-specific stroke	I64	436

### Information Sources

We searched for eligible articles in five databases (Medline, Embase, Scopus, Web of Science, and Cochrane Library), two grey literature sources (OpenGrey and Ethos), and, where available, the bibliographies of EHR databases from the date of inception to April 2019 in any language.

### Search Strategy

We searched medical subject heading terms and free-text (in the title and abstract) for the concepts of (1) CVD diagnoses, (2) EHRs, (3) Europe, and (4) validation. Search terms were developed for Medline and transcribed for the remaining databases (S1 Appendix). To identify any additional articles, we checked reference lists of eligible articles and relevant systematic reviews.

### Study Selection and Data Collection

Two reviewers (J.A.D. and R.M.) independently screened the titles and abstracts of all retrieved articles, followed by the full-text of articles deemed eligible in the first stage. Our published protocol details the full data collection process.^14^ Briefly, we extracted data using a pre-defined template (S2 Appendix) which we piloted using dual extraction for three studies, followed by further parallel extraction for 20% of studies, and completed by a single reviewer (J.A.D.) for the remaining studies.

### Risk of Bias in Individual Studies

We used a modified version of the Quality Assessment of Diagnostic Accuracy Studies 2 (QUADAS-2)^18^ tool to assess bias (S3 Appendix). As with our data extraction, two authors (J.A.D. and R.M.) piloted the tool for three studies, then independently assessed risk in a further 10% of studies, with the process completed by a single reviewer (J.A.D.).

### Synthesis of Results

We synthesized results with a narrative approach, grouping studies by acute CVD diagnosis (HF, ACS or stroke) and, where possible, subgroups of interest. Subgroups were; diagnosis type, definition, data source including diagnostic position and coding system, reference standard, and study population including time period, age and sex. For studies that reported validation estimates without confidence intervals (CIs), but included raw data, we calculated 95% CIs using the Wilson method for binomial proportions. We used the I^2^ statistic to assess heterogeneity between the sensitivity and positive predictive value (PPV) estimates, following the Cochrane thresholds.^19^ Heterogeneity assessment did not include specificity or negative predictive value (NPV), as few studies reported these measures. To investigate sources of heterogeneity, we compared I^2^ before and after removing studies at a high risk of bias and by the previously mentioned subgroups. We used the Stata metaprop command^20^ to calculate I^2^. Metaprop uses raw data rather than precalculated estimates; studies that reported sensitivity or PPV but not the data used to calculate were excluded from heterogeneity assessment.

### Risk of Bias Across Studies

We used the Grading of Recommendations, Assessment, Development, and Evaluation (GRADE) tool for diagnostic accuracy systematic reviews^21^ to summarise cross-study quality. Evidence was categorised as “high”, “moderate”, “low” or “very low” quality. See S4 Appendix for the reasons we rated quality down or up.

## Results

### Studies Included

**Figure 2:**
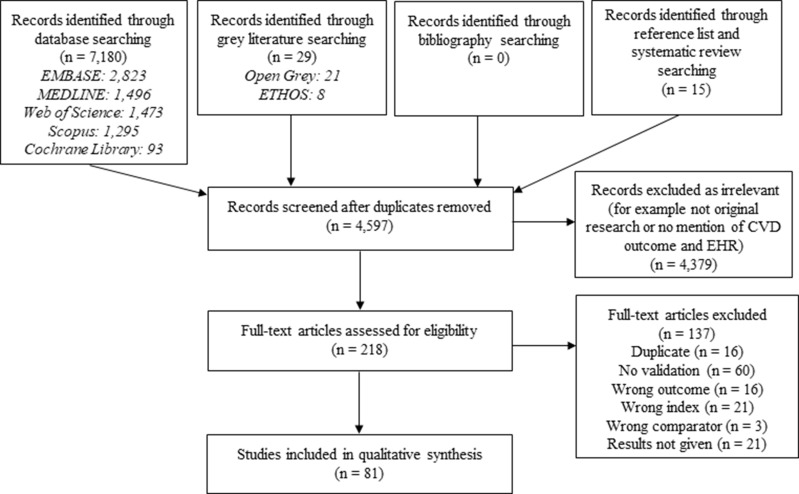
Flow diagram of study selection.

We identified 4595 studies, of which 218 were included in full-text review and 81 met eligibility criteria (Figure 2).

Study characteristics are summarized in S1 Table, results are displayed in S2 Table, Figures 3–8 and S1–6 Figs, additional subgroup results are described in S5 Appendix, QUADAS-2 results are in S3 Table, and our GRADE assessment is detailed in S4 Table.

**Figure 3:**
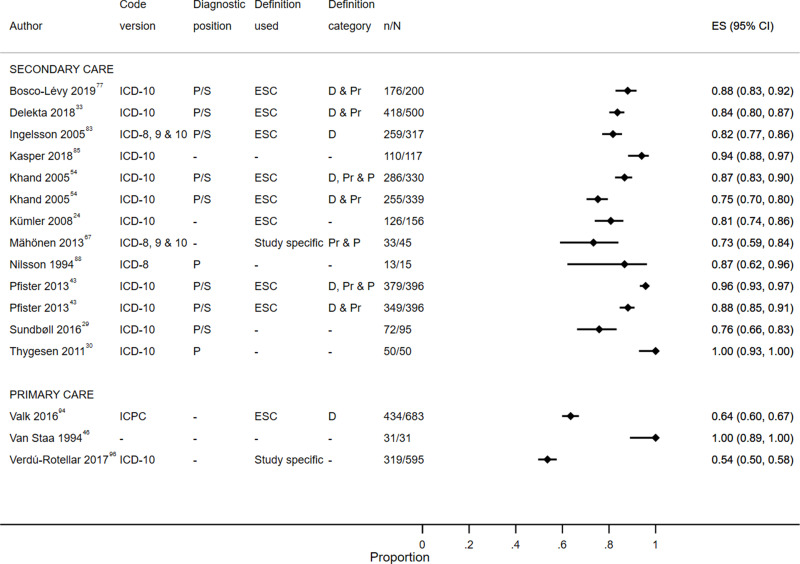
Positive predictive value for heart failure diagnoses from studies which reported the number of records confirmed positive and the total number of records.

**Abbreviations:** D, definite; D & Pr, definite and probable; D, Pr & P, definite, probable and possible; P, primary; P/S, primary or secondary.

**Figure 4:**
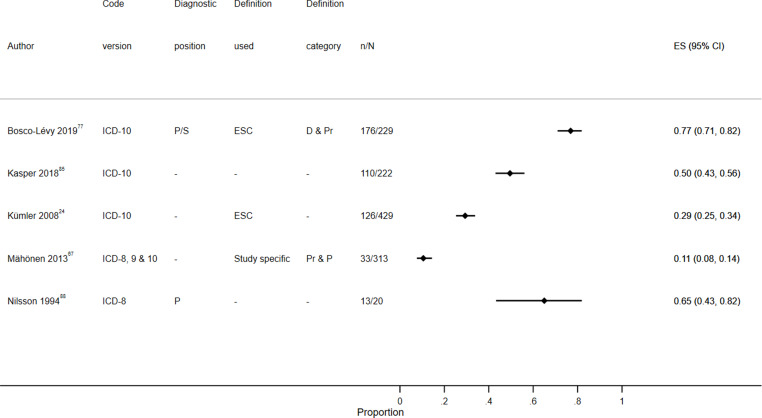
Sensitivity for heart failure diagnoses from studies which reported the number of records confirmed positive and the total number of records. **Abbreviations:** D & Pr, definite and probable; Pr & P, probable and possible; P, primary; P/S, primary or secondary.

**Figure 5:**
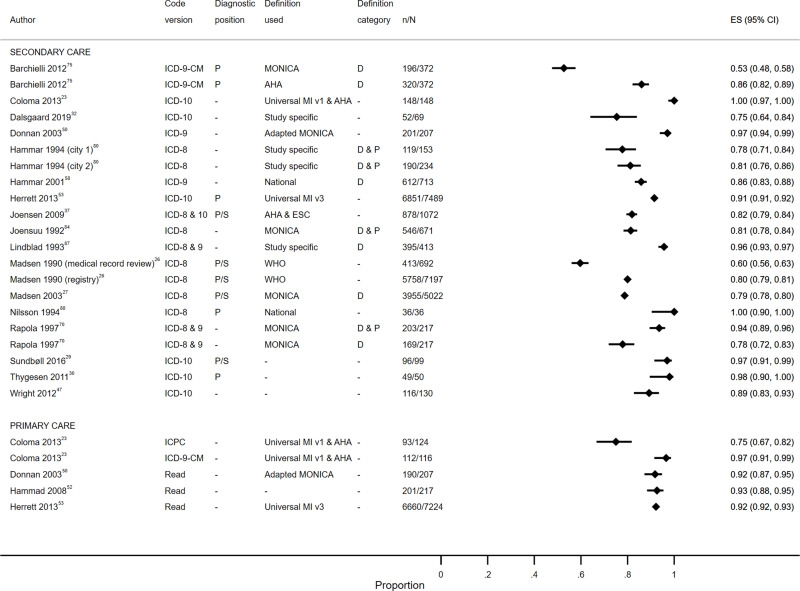
Positive predictive value for myocardial infarction diagnoses from studies which reported the number of records confirmed positive and the total number of records. **Abbreviations:** D, definite; D & P, definite and possible; P, primary; P/S, primary or secondary; RS, reference standard.

**Figure 6:**
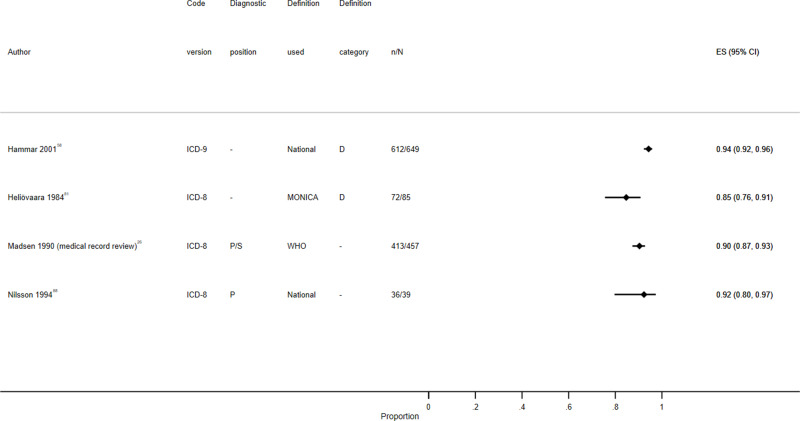
Sensitivity for myocardial infarction diagnoses from studies which reported the number of records confirmed positive and the total number of records. **Abbreviations:** D, definite; D & P, definite and possible; P, primary; P/S, primary or secondary; RS, reference standard.

**Figure 7:**
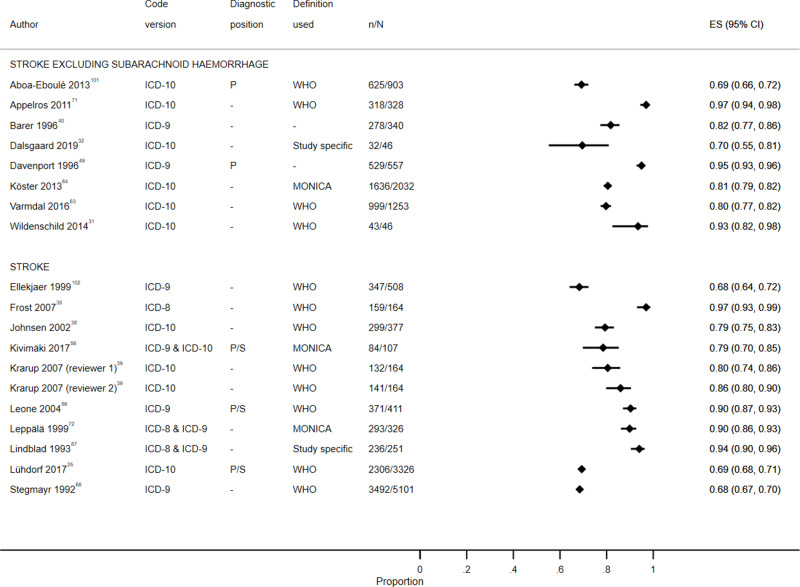
Positive predictive value for stroke diagnoses recorded in secondary care EHRs from studies which reported the number of records confirmed positive and the total number of records. **Abbreviations:** ES, effect size; P, primary; P/S, primary or secondary.

**Figure 8:**
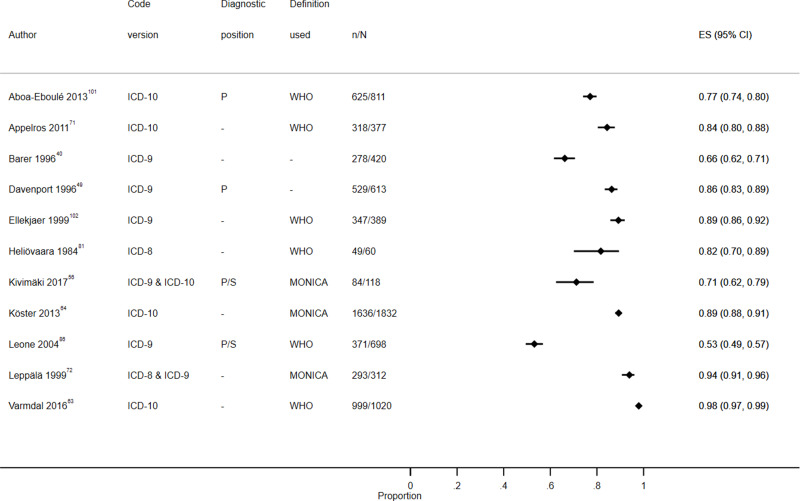
Sensitivity for stroke diagnoses recorded in secondary care EHRs from studies which reported the number of records confirmed positive and the total number of records. **Abbreviations:** ES, effect size; P, primary; P/S, primary or secondary.

### Study Characteristics

The 81 included studies validated EHRs from 11 different countries, most frequently Denmark (18 studies)^22^–^39^ and the UK (17 studies).^40^–^56^ Validation was the primary aim of all but 10 studies.^35^,^36^,^41^,^48^,^57^–^62^ Fourteen studies^26^,^27^,^31^,^63^–^73^ validated a vital registration system or disease registry in addition to the EHR. The records validated included data from 1969–2015. Where ICD coding was validated this covered versions 8–10. Sixty studies used medical record review as a reference standard.^22^,^23^,^25^–^39^,^42^,^43^,^45^,^46^,^49^,^50^,^54^,^55^,^57^–^63^,^69^,^72^,^74^–^96^ Twenty studies validated HF,^24^,^28^–^30^,^33^,^43^,^46^,^54^,^59^,^65^,^67^,^77^,^82^,^83^,^85^,^88^,^94^–^97^ 31 ACS^22^,^23^,^26^,^27^,^29^,^30^,^32^,^34^,^37^,^42^,^46^,^47^,^50^,^52^,^53^,^58^,^65^,^68^–^70^,^75^,^76^,^80^,^81^,^84^,^87^,^88^,^91^,^98^–^100^ and 41 stroke diagnoses.^25^,^31^,^32^,^35^,^36^,^38^–^41^,^44^,^45^,^47^–^49^,^51^,^55^–^57^,^60^–^64^,^66^,^71^–^74^,^78^,^79^,^81^,^86^,^87^,^89^–^93^,^98^,^101^,^102^

### Study Quality

Study quality was high for 54 (67%) of studies,^22^–^26^,^28^,^29^,^31^–^34^,^38^,^39^,^42^–^44^,^47^,^50^,^51^,^53^,^54^,^56^,^59^,^60^,^62^–^65^,^67^–^70^,^72^,^73^,^75^–^79^,^85^–^90^,^92^–^94^,^96^,^98^–^102^ medium for 19 (24%) studies^27^,^30^,^35^–^37^,^46^,^49^,^52^,^55^,^57^,^58^,^61^,^66^,^74^,^81^–^84^,^95^ and low for eight (10%) of studies.^40^,^41^,^45^,^48^,^71^,^80^,^91^,^97^ Studies were overall at low risk of bias in patient selection (76 low, 3 unclear, 2 high), index test (71 low, 10 high), and flow and timing (78 low, 3 unclear) domains and higher risk in the reference standard domain (36 low, 28 unclear, 17 high). Generally, reference standard methods and definitions were poorly described, and on occasion the reference standard was not independent of the EHR. Risk of bias was also higher in studies which validated primary care EHRs. HF validation studies had high quality in 14 (70%) studies, medium in five (25%) and low in one (5%). For ACS validation, quality was high for 21 (68%), medium for eight (26%) and low for two (6%) studies. In stroke validation studies, quality was high for 26 (63%), medium for nine (22%) and low for six (15%) studies.

### Heart Failure Study Characteristics

HF diagnoses were most extensively validated using EHR data from Denmark (five studies),^24^,^28^–^30^,^33^ the Netherlands (four studies),^59^,^65^,^94^,^95^ Sweden (three studies)^82^,^83^,^88^ and the UK (three studies).^43^,^46^,^54^ In addition, EHR data from Finland,^67^ France,^77^ Germany,^85^ Italy^97^ and Spain^96^ were validated in one study each. Fourteen studies validated secondary care EHRs^24^,^28^–^30^,^33^,^43^,^54^,^59^,^65^,^67^,^77^,^83^,^85^,^88^ and six studies validated primary care EHRs.^46^,^82^,^94^–^97^ Medical record review was used as the reference standard in all but three studies.^24^,^65^,^97^

### Heart Failure Validation Results

#### Overall

From the main validation result reported by each of the studies; sensitivity (available from nine studies)^24^,^46^,^65^,^67^,^77^,^82^,^85^,^88^,^95^ was ≥50% in six studies^46^,^77^,^82^,^85^,^88^,^95^ but >66% (range 11–100%) in only one study,^46^ PPV (19 studies)^24^,^28^–^30^,^33^,^43^,^46^,^54^,^59^,^65^,^67^,^77^,^83^,^85^,^88^,^94^–^97^ was ≥80% (range 54–100%) in all but five studies,^29^,^67^,^94^,^96^,^97^ specificity (three studies)^24^,^67^,^95^ was ≥95% in all studies, and NPV (three studies)^24^,^67^,^95^ was ≥84% (range 84–96%) in all studies.

#### Diagnosis Type

In the three studies that reported results for first diagnosis, the PPV range was 76–88%.^28^,^29^,^77^ One study compared the PPV for all diagnoses (84%) to first diagnosis (80%),^28^ and another study found the same PPV for first diagnosis and recurrent diagnosis (both 76%).^29^

#### Definition

In seven of the eight studies^24^,^28^,^33^,^43^,^54^,^77^,^83^,^94^ which used the ESC definition,^15^ the PPV was ≥80%. The study^94^ with the lower PPV of 64% was the only one to validate a primary care EHR. Other studies used; both Framingham^103^ and Boston^104^ criteria (one study,^59^ PPV 80–81%), the American College of Cardiology (ACC)/American Heart Association (AHA) definition^105^ (one study,^97^ PPV 55%), or study-specific definitions (three studies,^67^,^95^,^96^ PPV 54–83%). An overview of the definitions used by the studies is presented in S6 Appendix.

Seven studies reported classification criteria; the PPV for definite HF ranged between 61–82%,^33^,^43^,^54^,^77^,^83^ including both definite and probable HF increased the PPV to 73–88%^33^,^43^,^54^,^77^,^83^,^94^ and the two studies which additionally included possible HF reported high PPV as 87%^54^ and 96%.^43^

#### Diagnostic Position

Six studies^29^,^33^,^43^,^54^,^77^,^83^ reported HF recorded in any diagnostic position (PPV 76–96%) and two studies^30^,^88^ only included primary position (PPV 87% and 100%). Three studies,^33^,^77^,^83^ which validated any position, also included breakdowns by primary (PPV 88–96%) and secondary (PPV 66–84%) positions.

#### Coding System

Twelve studies validated ICD-10,^24^,^28^–^30^,^33^,^43^,^54^,^67^,^77^,^82^,^83^,^96^ with all but one^83^ reporting results specifically for this version of ICD (PPV 78–99%). Six studies^24^,^33^,^43^,^77^,^82^,^96^ validated I50; two studies of primary care EHRs reported lower validity estimates (PPV 54%^96^ and sensitivity 66%)^82^ compared to four studies of secondary care EHRs (PPV 81–96%,^24^,^33^,^43^,^77^ and sensitivity 29%^24^ and 64%).^77^ Five studies included a broader range of ICD-10 codes, all of which differed. The estimates for ICD-10 codes were no higher than those for ICD-8 (PPV 87%),^67^,^83^,^88^ ICD-9 (PPV 79–97%),^59^,^65^,^67^,^83^ or combinations of the three ICD systems (PPV 73–82%).^67^,^83^ Two studies validated ICPC K77 in primary care EHRs (PPV 64%^94^ and 83%^95^).

### Acute Coronary Syndrome Study Characteristics

Similar to HF, ACS diagnoses were most frequently validated using EHR data from Denmark (nine studies),^22^,^23^,^26^,^27^,^29^,^30^,^32^,^34^,^37^ followed by Finland (seven studies),^68^–^70^,^81^,^84^,^99^,^100^ the UK (six studies)^42^,^46^,^47^,^50^,^52^,^53^ and Sweden (4 studies).^58^,^80^,^87^,^88^ Two studies validated data in each of Italy,^23^,^75^ the Netherlands,^23^,^65^ and Spain,^91^,^98^ and a final study used data from France.^76^ Twenty-six of the studies validated a secondary care EHR,^22^,^26^,^27^,^29^,^30^,^32^,^34^,^37^,^42^,^47^,^58^,^65^,^68^–^70^,^75^,^76^,^80^,^81^,^84^,^87^,^88^,^91^,^98^–^100^ three studies validated both a primary and secondary care EHR^23^,^50^,^53^ and two studies validated a primary care EHR.^46^,^52^

Four studies^22^,^37^,^68^,^76^ presented overall ACS results, of which one study^68^ included an additional breakdown for MI and two studies^37^,^76^ included unstable angina and MI, one of which also included cardiac arrest.^37^ A further two studies^29^,^65^ did not report results for ACS overall but did include both unstable angina and MI. The remaining 25 studies solely validated MI diagnoses.^23^,^26^,^27^,^30^,^32^,^34^,^42^,^46^,^47^,^50^,^52^,^53^,^58^,^69^,^70^,^75^,^80^,^81^,^84^,^87^,^88^,^91^,^98^–^100^

### Acute Coronary Syndrome Validation Results

#### Overall

For ACS, three studies^33^,^37^,^76^ reported one main PPV (range 66–87%), while results presented by Pajunen et al^68^ were broken down by age, sex and time period, with sensitivity of 66–87% and PPV of 63–86%.

#### Diagnosis Type

The PPV for unstable angina varied; with low values of 20%^76^ and 27.5%^37^ in two studies and higher values of 78%^65^ and 88%^29^ in the other two studies. Sensitivity was only reported by one study,^65^ at 53%. For MI, the main validation result for sensitivity (11 studies)^26^,^27^,^34^,^42^,^46^,^50^,^58^,^65^,^81^,^88^,^98^ was ≥80% in all but one study^42^ (range 56–97%), and six^26^,^27^,^34^,^58^,^88^,^98^ >90%. PPV (24 studies)^23^,^26^,^27^,^29^,^30^,^32^,^34^,^37^,^42^,^46^,^47^,^50^,^52^,^53^,^58^,^65^,^70^,^75^,^76^,^80^,^84^,^87^,^88^,^98^ was ≥80% (range 42–100%) in all but three studies^27^,^32^,^34^ with 12^23^,^29^,^30^,^42^,^50^,^52^,^53^,^65^,^87^,^88^,^98^ ≥90%. Three studies^34^,^42^,^98^ reported specificity (range 93–100%) and two^34^,^98^ included NPV (range 82–100%).

Four studies^29^,^32^,^37^,^84^ reported the PPV for first MI, with estimates of 75–97%, and one study^29^ also included recurrent MI with a PPV of 88% compared to 97% for first MI.

#### Definition

Varying MI definitions were used (S6 Appendix). Most frequently (nine studies)^26^,^27^,^50^,^70^,^75^,^81^,^84^,^99^,^100^ the World Health Organization (WHO) Monitoring trends and determinants in cardiovascular disease (MONICA) definition106 was used, with variable PPV estimates of 53–96% obtained. Two studies compared MONICA to another MI definition; one^75^ showed MONICA-defined definite MI had a substantially lower PPV than AHA/ESC-defined^16^ definite MI (53% vs 86%), while the other^84^ also showed a lower PPV for MONICA compared to “normal clinically defined MI” but with a smaller difference (81% vs 89%). One further study used the AHA/ESC definition^37^ (PPV 82%). The universal definition^107^ was used in a study^23^ which included EHR data from three countries, with PPVs of 75–100%. Three studies used the third universal definition,^108^ one^76^ of which combined it with the earlier universal definition (PPV 85%). In another^53^ PPVs of 92% with obtained for the primary and secondary care EHRs validated. The third^34^ validated MI diagnoses recorded for patients with drug-eluting coronary stents, the PPV was 42% for all admission and 73% for acute admissions.

#### Diagnostic Position

Of the 10 studies which reported the diagnostic position used to validate MI diagnoses, five^26^,^27^,^29^,^34^,^68^ used any diagnostic position (PPV 42–97%) and five^30^,^75^,^76^,^88^,^98^ primary position (PPV 53–100%). One study^27^ which validated any position (PPV 79%) also included a breakdown by primary position (PPV 80%) and another study^29^ included breakdowns by primary (PPV 99%) and secondary positions (PPV 80%).

#### Coding System

Ten studies validated ICD-10 coded MI, eight reported results specifically for ICD-10.^23^,^29^,^30^,^32^,^34^,^47^,^53^,^76^ Four studies validated ICD-10 I21 with PPV ≥85% (range 42–100%)^23^,^29^,^34^,^76^ in all but one.^34^ Two studies included I21-I23 and reported high PPVs of 92%^53^ and 98%;^30^ however, the latter study was small in size (50 patients). One study validated I21-I22 (PPV 89%)^47^ and another I21-I24 (PPV 75%).^32^ The estimates for ICD-10 codes were no higher than those for ICD-8 (PPV 79–100%),^26^,^27^,^80^,^84^,^88^ ICD-9 (86–100%),^42^,^50^,^58^,^65^,^75^,^98^ or combinations of three ICD systems (PPV 82–96%).^37^,^87^ Of the studies to validate data in primary care, one^23^ included IPCI K75 code (PPV 75%) and three^50^,^52^,^53^ validated Read coding in the UK (PPV 91–93%).

#### Reference Standard

The PPV for MI diagnoses varied between 53–100% when medical record review was the reference standard (20 studies)^22^,^23^,^26^,^29^,^30^,^32^,^37^,^42^,^46^,^50^,^58^,^69^,^70^,^75^,^76^,^80^,^84^,^87^,^88^,^91^ and 89–93% when a registry was used.^26^,^27^,^53^,^68^,^98^–^100^ One study^34^ used medical record review after comparing EHR and registry results (PPV 42%). Two studies used a GP questionnaire (PPV 89% and 93%),^47^,^52^ and one study used a local cardiology database (PPV 97%).^65^

### Stroke Study Characteristics

Stroke diagnoses were most frequently validated in UK EHRs, with 10 studies conducted,^40^,^41^,^44^,^45^,^47^–^49^,^51^,^55^,^56^ followed by Denmark (seven studies),^25^,^31^,^32^,^35^,^36^,^38^,^39^ Sweden (5 studies)^60^,^64^,^66^,^71^,^87^ and Italy (4 studies).^74^,^86^,^90^,^93^ Data from Finland,^72^,^73^,^81^ France,^78^,^79^,^101^ Norway,^63^,^89^,^102^ and Spain^62^,^91^,^98^ were validated in three studies each. A further two studies validated EHR data from the Netherlands^57^,^61^ and one from the Czech Republic.^92^ All but three studies^41^,^44^,^48^ validated secondary care EHRs.

Twenty-eight studies presented validation estimates for overall stroke (including both ischaemic and haemorrhagic).^25^,^31^,^32^,^35^,^38^–^41^,^44^,^45^,^48^,^49^,^56^,^60^,^63^,^64^,^66^,^71^–^73^,^81^,^86^,^87^,^91^,^92^,^98^,^101^,^102^ Ischaemic stroke was assessed in 18 studies,^25^,^32^,^38^,^39^,^47^,^57^,^62^,^72^–^74^,^78^,^79^,^86^,^90^,^92^,^93^,^101^,^102^ in all but four studies^62^,^74^,^79^,^90^ this was done as a subgroup analysis after validating overall stroke. Similarly, haemorrhagic stroke was assessed by 21 studies; two reported results for overall haemorrhagic stroke^32^,^51^ with this the main focus of one study,^51^ 17 studies reported results for ICH as a subgroup analysis^25^,^38^,^39^,^47^,^51^,^55^,^57^,^72^,^73^,^78^,^86^,^87^,^89^,^92^,^93^,^101^,^102^ and 18 studies reported results for SAH^25^,^36^,^38^,^39^,^47^,^51^,^55^,^61^,^72^,^73^,^78^,^81^,^86^,^87^,^89^,^92^,^93^,^102^ with this being the main result in two studies.^36^,^61^

### Stroke Validation Results

#### Overall

For overall stroke, sensitivity (15 studies)^31^,^40^,^45^,^49^,^56^,^63^,^64^,^71^,^73^,^81^,^86^,^91^,^98^,^101^,^102^ was ≥80% (range 33–97%) in seven studies^49^,^63^,^64^,^71^,^73^,^81^,^102^ and ≥70% in 11 studies. PPV (27 studies)^25^,^31^,^32^,^35^,^38^–^41^,^45^,^48^,^49^,^56^,^60^,^63^,^64^,^66^,^71^–^73^,^81^,^86^,^87^,^91^,^92^,^98^,^101^,^102^ was ≥80% (range 20–97%) in 19 studies.^31^,^35^,^39^–^41^,^45^,^48^,^49^,^60^,^63^,^64^,^71^,^72^,^81^,^86^,^87^,^92^,^98^ Nine of the studies^31^,^32^,^40^,^49^,^60^,^63^,^64^,^71^,^101^ did not include codes to validate SAH, three of which had stated this in their inclusion criteria.^40^,^71^,^101^ Excluding these studies did not affect the sensitivity (53–89%) or PPV (68–97%). Specificity and NPV, reported by five studies, were 99–100%^49^,^56^,^63^,^98^ other than one study^31^ which obtained a specificity of 96% and NPV of 72%.

#### Diagnosis Type

Three studies^56^,^64^,^101^ included first and recurrent overall stroke with sensitivity from 71–89% and PPV 69–81%, while three studies^32^,^71^,^73^ also included only first stroke for which sensitivity was 85–89% and PPV 70–97%.

For ischaemic stroke, the main sensitivity reported (6 studies)^74^,^79^,^81^,^86^,^90^,^102^ was ≥66% in all but one^86^ study (range 37–82%). Fourteen studies^25^,^32^,^38^,^47^,^57^,^62^,^72^,^74^,^78^,^79^,^86^,^90^,^92^,^102^ included one main PPV of 66–96%. One study^101^ classified results separately for cardiac embolism, large artery atherosclerosis, lacunar infarct and ischaemic stroke of other aetiology. Sensitivity and PPV were highest in the cardiac embolism classification (83% and 87%, respectively) and lowest for other aetiology (67% and 35%, respectively). For ICH, the main sensitivity reported was 59–98% (4 studies)^73^,^86^,^101^,^102^ and main PPV 55–96% (15 studies).^25^,^38^,^39^,^47^,^51^,^55^,^57^,^72^,^73^,^78^,^86^,^87^,^92^,^101^,^102^ The sensitivity of SAH diagnoses was 35–92% (4 studies)^73^,^81^,^86^,^102^ and PPV was 42–96% (18 studies).^25^,^36^,^38^,^39^,^47^,^51^,^55^,^61^,^72^,^73^,^78^,^81^,^86^,^87^,^89^,^92^,^93^,^102^

#### Definition

Stroke was defined in 22 of the 41 studies, 13^25^,^31^,^35^,^38^,^39^,^63^,^66^,^71^,^81^,^86^,^90^,^92^,^101^,^102^ used the WHO definition109 (sensitivity 53–97%^63^,^71^,^86^,^101^,^102^ and PPV 68–97%),^25^,^35^,^38^,^39^,^63^,^66^,^71^,^81^,^86^,^92^,^101^,^102^ seven^56^,^60^,^62^,^64^,^72^,^74^,^93^ used MONICA^110^ (sensitivity 71–89%^56^,^64^ and PPV 79–92%),^56^,^60^,^64^,^72^ and two^32^,^87^ defined stroke specifically for their study (PPV 70% and 91%). The stroke definitions used are summarized in S6 Appendix.

#### Diagnostic Position

For overall stroke diagnoses recorded in any diagnostic positions, sensitivity ranged from 53–97%^56^,^63^,^86^ and PPV from 69–90%.^25^,^56^,^63^,^86^ In comparison, results only for primary position were 67–86% for sensitivity and 69–95% for PPV.^49^,^63^,^73^,^98^,^101^

#### Coding System

Thirteen studies validated ICD-10 (PPV 20–97%,^31^,^32^,^38^,^39^,^45^,^47^,^55^,^60^,^63^,^64^,^71^,^78^,^92^ sensitivity 76–97%).^45^,^63^,^64^,^71^,^101^ Four studies^31^,^63^,^64^,^71^ which excluded SAH from the stroke definition validated ICD-10 I61, I63 and I64 (sensitivity 89–97% and PPV 79–97%). Aboa-Eboule et al^101^ additionally included G46 in their definition (sensitivity 77% and PPV 69%) while Dalsgaard et al^32^ validated I61-I65 (PPV 70%). In comparison, Holmqvist et al^60^ only included I61 and I63, and obtained PPV estimates of 92% and 89% in people with and without rheumatoid arthritis, respectively. Three studies^38^,^39^,^92^ which included SAH in the stroke definition validated I60, I61, I63 and I64 (PPV 79–86%) and one^45^ additionally included I62 (PPV 96%). The estimates for ICD-10 codes were no higher than those for ICD-8 codes (sensitivity 82%),^81^ ICD-9 (PPV 20–95%,^40^,^49^,^66^,^86^,^91^,^93^,^98^,^102^ sensitivity 33–89%),^40^,^49^,^86^,^91^,^98^,^102^ or combinations of three ICD systems (PPV 79–97%,^35^,^72^,^73^,^87^ sensitivity 71–85%).^73^

Seven studies validated ICD-10 I63 for ischaemic stroke diagnosis (PPV 78–96%).^25^,^32^,^38^,^47^,^78^,^79^,^92^ One study^73^ used a broad (ICD-9433, 434, 436 and ICD-10 I63, I64) and narrow range of codes (ICD-9433, 434 and ICD-10 I63) to define ischaemic stroke, with similar sensitivity (82% vs 81%) and PPV (84% vs 83%). One other study^74^ reported results by ICD-9 codes 443*1 and 434*1 (PPV 86% and 90%, respectively). Six studies^25^,^38^,^55^,^78^,^89^,^92^ validated ICD-10 I61, with another two^39^,^101^ presumed to have also validated this code, for ICH (PPV 66–96%) and a further three studies^86^,^93^,^102^ validated ICD-9431 (PPV 71–78%). For SAH, eight studies^25^,^38^,^39^,^47^,^55^,^78^,^89^,^92^ validated ICD-10 I60 with PPV >90% in half of the studies (range 46–96%), four studies^61^,^86^,^93^,^102^ validated ICD-9430 (PPV 42–95%), one study^81^ validated ICD-8430 (PPV 85%) and two studies^72^,^87^ validated both versions for 430 (PPV 78–79%).

#### Reference Standard

In the 17 studies^25^,^31^,^32^,^35^,^38^,^39^,^45^,^55^,^56^,^60^,^63^,^72^,^79^,^86^,^87^,^91^,^92^ which used medical record review as the reference standard to validate overall stroke diagnoses, the PPV was ≥79% (range 20–97%) in all but four studies.^25^,^31^,^32^,^91^ A further eight studies used a registry reference standard (PPV 88–97%).^40^,^64^,^66^,^71^,^73^,^98^,^101^,^102^

### Heterogeneity

We were able to assess the heterogeneity between the main PPV reported in; 14 studies with 16 estimates of HF (I^2^=97.0%), 18 studies with 26 estimates of MI (I^2^=98.5%), and 19 studies with 20 estimates of stroke (I^2^=97.9%) diagnoses. Additionally, we assessed heterogeneity between the main sensitivity for; six studies of HF (I^2^=98.6%), four of MI (I^2^=74.3%), and 11 of stroke (I^2^=98.8%) diagnoses. Heterogeneity between the estimates was considerable, at more than >95% in all cases other than sensitivity estimates for MI. Furthermore, heterogeneity remained considerable after removal of studies at a high risk of bias.

### Overall Strength of Evidence

GRADE showed that cross-study quality was very low for all HF outcomes (sensitivity and PPV in secondary care EHRs and PPV in primary care EHRs), low for MI sensitivity and PPV in secondary care EHRs and moderate for PPV in primary care EHRs, and very low for stroke sensitivity in secondary care EHRs and PPV in primary care EHRs and moderate for PPV in secondary care EHRs.

## Discussion

### Summary of Findings

Our systematic review suggests that the sensitivity of coded data in European EHRs for HF diagnoses is low at ≤66% in all but one study. There was also wide variation in stroke sensitivity estimates, with only half of studies ≥80%, although three-quarters were ≥70%. The sensitivity of ACS was higher at ≥80% in the vast majority of studies. The majority of studies which validated ACS diagnosis did so specifically for MI.

The PPV of all diagnoses was ≥80% in the majority of studies; two-thirds for HF (nearly three-quarters for secondary care EHRs), nearly three-quarters for MI, and 70% of stroke validation studies. Where subtypes were validated, PPV was ≥80% for four-fifths of ischaemic stroke diagnoses but only 44% of ICH and SAH diagnoses.

The specificity and NPV were also high where available (three HF studies, three MI studies and five stroke studies). However, as most studies only included patients with the diagnosis of interest recorded in the EHR and reference standard, the results presented were mostly limited to sensitivity and PPV.

Both PPV and NPV are impacted by disease prevalence, with lower estimates for rare conditions.^111^ Our systematic review focused on Europe, drawing studies from 11 countries. Age-standardized prevalence of CVD in these countries is between 5000–6500 per 100,000, other than the Czech Republic (~8700 per 100,000) which only contributed one study.^2^ Therefore, prevalence differences should have limited impact on our comparison of validity estimates between geographies. The prevalence of CVD increases with age, but we did not find any systematic difference in results between studies with younger or older populations.

The low sensitivity of HF diagnoses we identified is consistent with a previous systematic review validating HF diagnoses in administrative data, which identified three European studies.^11^ Twelve more studies have since been published and included in our review. These more recent findings, however, do not suggest any improvement in the quality of data over time. This is perhaps unsurprising given the range of clinical aetiology and presentation. The high proportion of studies we found to have a PPV of <80% for stroke diagnoses appeared more substantial than in previous systematic reviews.^9^,^12^ We identified 15 new studies which were not included in these previous reviews.^25^,^32^,^45^,^51^,^56^,^57^,^61^–^63^,^74^,^78^,^89^,^91^,^92^,^98^ Our results for sensitivity and PPV of MI diagnoses are consistent with previous reviews,^8^,^10^ and identified five^29^,^32^,^34^,^76^,^98^ new MI validation studies with variable results.

There was substantial heterogeneity between the sensitivity and PPV estimates for all three acute CVD diagnoses. Heterogeneity was likely because studies differed in multiple ways; for example, even among studies which used medical record review as the reference standard, differences in study time period impacted upon the ICD version used. The heterogeneity caused by variable methods was highlighted in previous systematic reviews of atrial fibrillation and dementia diagnoses recorded in routine health data.^112^,^113^

### Defining Diagnosis in the EHR

We were most interested in the results of ICD-10 validation, as this is the latest ICD coding system which is widely used in Europe and elsewhere. In McCormick et al’s^10^ review of MI diagnoses in administrative data, the authors noted a lack of ICD-10 validation with only three studies identified, whereas our review identified 10. Nevertheless, even within ICD-10, combinations of codes used, and therefore their validity, differed, which highlights the importance of tailoring codes to each research question. Codes are arguably even more important when using other, more complex coding systems such as Read codes, which are used in UK primary care data and can generate vast numbers of codes for every clinical condition.

### Defining Diagnosis in the Reference Standard

There is no single recommended gold standard to determine the validity of EHR data.^114^ Nearly three-quarters (74%) of studies used medical records; more frequently for HF diagnoses (85%) than ACS (71%) or stroke (68%). This difference may be due to availability of MI and stroke registries, used in 26% and 22% of studies, respectively. No differences in the performance of the reference standard methods were discernable, probably due to heterogeneity.

Criteria to define CVD, especially MI, have been refined over time, driven by the development of more sensitive and specific biomarkers, and more precise imaging techniques.^100^ However, we did not identify any temporal trends in the accuracy of MI recording, again likely due to overall study heterogeneity.

When validating HF, which can vary in clinical aetiology and presentation, clarity on the criteria used to define, with explicit classification of acute and chronic HF along with ejection fraction would benefit understanding of results.

### Comparing and Combining Data Sources

Only 14 (17%) studies validated primary care systems, more than half of which were in the UK. Using primary care EHRs may be beneficial for research into conditions such as HF which are frequently managed in primary care; in our study, 30% of HF EHR validation studies used primary care data, compared to 16% for ACS and 7% for stroke studies. For acute severe conditions resulting in hospitalization, secondary care records should be the most reliable data source. Where possible, the use of linked data to increase the ascertainment of acute CVD events should be considered.

### Implications for Future Research

EHR-based research is a growing field – widely used in observational analyses and increasingly employed in trials.^115^ Researchers should consider the level of validity necessary for their own CVD outcome definition. When a composite outcome, such as MACE, is used researchers may need to address differing sensitivity in the individual components of the outcome. In studies which investigate CVD incidence, a sensitive definition is particularly important. For example, EHR data are being used for rapid COVID-19 pandemic analyses such as; the impact the virus has in those with CVD, CVD as an outcome after infection with the virus, and excess death estimates.^116^ It is important that these rapid analyses consider the validity of the data and definitions used. Conversely, in a pragmatic trial recruitment, a specific definition is likely more important than a sensitive one.

### Strengths and Limitations

Our systematic review provides a comprehensive and up-to-date evaluation of the validity of acute CVD diagnoses in European EHRs, conducted without language or time restrictions using a broad search strategy. Two independent reviewers performed our study selection, and native speaking collaborators translated foreign language articles. Similar to other systematic reviews of validation studies, we repurposed the QUADAS-2 risk of bias tool developed for diagnostic test accuracy. Additionally, we followed the diagnostic test accuracy GRADE methodology to assess the overall evidence base.

Our work is not without limitations. Firstly, only one reviewer completed full data extraction and risk of bias assessment due to resource constraints, although a sample of 20% of studies had data dual extracted. Secondly, we limited our study to Europe, so theoretically our results are only generalizable to European countries. All previous systematic reviews^8^–^12^ on the validity of acute CVD diagnoses included both EHRs and claim-based systems, while most studies included in each of these reviews were from North America. From these existing reviews, it was unclear if the validity of EHRs differed to claims-based datasets, which reflect payments related to medical care given. Despite this, we obtained similar results to the previous reviews. Thirdly, our review focused on acute CVD events so excluded results from studies that validated broader diagnoses of ischaemic heart disease or cerebrovascular disease, which again limits generalizability to these specific conditions.

### Recommendations

For ACS and stroke diagnoses, most sensitivity and PPV results were reasonably high, providing confidence in the use of European EHR data for research into these conditions. However, there was considerable heterogeneity between studies. Sensitivity for HF diagnoses was low, and our GRADE assessment found very low quality for all HF outcomes. For studies of HF, we strongly recommend either validating the definition or referring to existing validation studies to develop the case definition. New validation studies of HF diagnoses should report whether the diagnoses validated are for acute or chronic presentation and HF with reduced ejection fraction or preserved ejection fraction. These principles are also applicable to future ACS and stroke validation studies. Identifying specific stroke subtypes can be difficult; analysis of all stroke subtypes combined is preferable.

## Conclusions

Our review on the accuracy of HF, ACS and stroke diagnoses in European EHRs should guide researchers in their selection of data sources and CVD definitions for epidemiological studies. Generally, the data assessed was of reasonable quality. However, it is difficult to summarize validity given the heterogeneity between studies. Where possible, researchers should validate data before use or carefully interpret the results of previous validation studies to consider the impact validity has on research findings. Additionally, the use of linked data will bolster quality.
